# A New Mouse Model for Female Genital Schistosomiasis

**DOI:** 10.1371/journal.pntd.0002825

**Published:** 2014-05-01

**Authors:** Monica L. Richardson, Chi-Ling Fu, Luke F. Pennington, Jared D. Honeycutt, Justin L. Odegaard, Yi-Ju Hsieh, Olfat Hammam, Simon L. Conti, Michael H. Hsieh

**Affiliations:** 1 Departments of Ob/Gyn and Urology, Female Pelvic Medicine and Reconstructive Surgery, Stanford University School of Medicine, Palo Alto, California, United States of America; 2 Department of Urology, Stanford University School of Medicine, Palo Alto, California, United States of America; University of Manchester, United Kingdom

## Abstract

**Background:**

Over 112 million people worldwide are infected with *Schistosoma haematobium*, one of the most prevalent schistosome species affecting humans. Female genital schistosomiasis (FGS) occurs when *S. haematobium* eggs are deposited into the female reproductive tract by adult worms, which can lead to pelvic pain, vaginal bleeding, genital disfigurement and infertility. Recent evidence suggests co-infection with *S. haematobium* increases the risks of contracting sexually transmitted diseases such as HIV. The associated mechanisms remain unclear due to the lack of a tractable animal model. We sought to create a mouse model conducive to the study of immune modulation and genitourinary changes that occur with FGS.

**Methods:**

To model FGS in mice, we injected *S. haematobium* eggs into the posterior vaginal walls of 30 female BALB/c mice. A control group of 20 female BALB/c mice were injected with uninfected LVG hamster tissue extract. Histology, flow cytometry and serum cytokine levels were assessed at 2, 4, 6, and 8 weeks post egg injection. Voiding studies were performed at 1 week post egg injection.

**Results:**

Vaginal wall injection with *S. haematobium* eggs resulted in synchronous vaginal granuloma development within 2 weeks post-egg injection that persisted for at least 6 additional weeks. Flow cytometric analysis of vaginal granulomata revealed infiltration by CD4^+^ T cells with variable expression of the HIV co-receptors CXCR4 and CCR5. Granulomata also contained CD11b^+^F4/80^+^ cells (macrophages and eosinophils) as well as CXCR4^+^MerTK^+^ macrophages. Strikingly, vaginal wall-injected mice featured significant urinary frequency despite the posterior vagina being anatomically distant from the bladder. This may represent a previously unrecognized overactive bladder response to deposition of schistosome eggs in the vagina.

**Conclusion:**

We have established a new mouse model that could potentially enable novel studies of genital schistosomiasis in females. Ongoing studies will further explore the mechanisms by which HIV target cells may be drawn into FGS-associated vaginal granulomata.

## Author Summary

Over 112 million people worldwide are infected with *Schistosoma haematobium* worms. *S. haematobium* eggs tend to be deposited in the tissue of pelvic organs such as the urinary bladder, lower ureters, cervix and vagina. Key sequelae include hematuria, dysuria, urinary frequency, and an increased risk of bladder cancer. This form of schistosomiasis can also cause dyspareunia, vaginal bleeding, pruritis, and granulomata that appear as tumors in the female genital tract. Collectively, these signs and symptoms are termed female genital schistosomiasis (FGS). Recent studies suggest that FGS occurs more commonly in girls and women with HIV, suggesting that it may be a risk factor for becoming HIV-infected. Unfortunately, the pathophysiology of this co-infection is not well understood. A lack of an experimentally manipulable model has contributed to the paucity of research focusing on this parasite. We have circumvented the barriers to natural *S. haematobium* oviposition in the mouse by directly microinjecting parasite eggs into the vaginal mucosa. The injection of *S. haematobium* ova appears to trigger vaginal inflammation and scarring infiltration by leukocytes expressing HIV co-receptors, and increased urinary frequency. Our approach may provide a representative animal model that could contribute to new opportunities to better understand the basic molecular and cellular immunology of female genital schistosomiasis.

## Introduction

An estimated 240 million humans worldwide have schistosomiasis, an infection by *Schistosoma* worms of various species [1]. Human infection begins when aquatic cercariae found in contaminated water penetrate intact skin. Once in the human host, these cercariae migrate into the circulation as schistosomula where in the portal vein they mature into adult worm mating pairs and then migrate to various venous plexi [2]. Three species of *Schistosoma* are primarily responsible for human disease, and *Schistosoma haematobium* contributes to over half of all cases of schistosomiasis [3]. With *S. haematobium* infection, worms can live and lay eggs for an average of 3.4 years [4]. When *S. haematobium* eggs deposit along the female genitourinary tract such as the urinary bladder, lower ureters, cervix and vagina, girls and women can experience hematuria, dysuria, urinary frequency, and an increased risk of bladder cancer [5]. However, *S. haematobium* infection is postulated to also cause dyspareunia, vaginal bleeding, pruritis, and giant granulomata that appear as tumors in the female genital tract [6]. Collectively, these signs and symptoms are termed female genital schistosomiasis (FGS) [7].

Recent studies suggest that FGS may cause women to be more susceptible to human immunodeficiency virus (HIV) infection [8]–[10] and those girls and women with FGS may have a 3-fold increased risk of contracting HIV [11]. Unfortunately, the pathophysiology of this co-infection is not well understood. Several studies have indicated, however, that other female genital infections, such as syphilis, human papilloma virus, and chlamydia, may increase the risk of HIV transmission [12], [13]. Genital infections that produce ulcers or vaginal discharge likely have the greatest impact on HIV shedding. This may be due to high concentrations of leukocytes in the genital tract, for example, during gonorrheal or chlamydial infections, that thereby lead to greater HIV shedding [14]. Syphilis is also associated with increased HIV shedding in the blood as well as genital tract [15]. Clinical features of FGS, including vascularized, “sandy patches” of disrupted vaginal mucosa which are susceptible to contact bleeding, likely promote viral transmission through sexual contact [9], [16], [17]. These lesions arise from an inflammatory response to deposited *S. haematobium* eggs, and contain inflammatory infiltrates, which may provide the optimal milieu for HIV transmission [18]. Eggs can trigger a significant immune response, including primarily Th2-skewed systemic immune deviation [19], [20] as well as the formation of egg-based granulomata. [19], [20].

Accordingly, an additional hypothesis for the increased HIV susceptibility of *S. haematobium*-infected girls and women (besides contact bleeding of genital lesions) postulates that *S. haematobium* infection results in systemic immune deviation which renders affected individuals more vulnerable to HIV infection. A third hypothesis for the enhanced HIV susceptibility of girls and women with FGS is that the close proximity of large numbers of granuloma-associated CD4^+^ T cells, macrophages, and dendritic cells (so-called HIV target cells) to infected genital tissues creates a convenient portal for HIV entry [21].

Currently there are no relevant animal models to study FGS-related pathology. Many questions remain regarding the mechanisms responsible for the genitourinary symptoms and possible increased rates of HIV transmission associated with FGS. Knowing the kinetics of how rapidly HIV target cells accumulate in FGS lesions is directly relevant to HIV prevention strategies for women and girls at risk of HIV. This in turn may drive the development of therapeutic interventions capable of limiting the immune and tissue pathology responsible for FGS-related sequelae. Unfortunately, natural transdermal infection of mice with *S. haematobium* cercariae results in hepatoenteric disease and very little if any pelvic organ pathology [22], [23]. Since the immune response is primarily directed against *S. haematobium* eggs, and not as prominently to other stages of the parasite lifecycle, we previously developed a mouse model of *S. haematobium* egg-induced bladder disease by direct injection of *S. haematobium* eggs into the mouse bladder wall [24]. This model recapitulates multiple aspects of human urinary schistosomiasis-associated bladder disease, including urinary frequency, hematuria, granuloma formation, and systemic immune responses.

Although, akin to oviposition in the bladder wall, the morbidity associated with FGS infection is strongly associated with egg deposition into the vagina and cervix, it is currently unclear whether oviposition alone, in the absence of adult worms, is sufficient to induce vaginal pathology. This is relevant to girls and women who have cleared *S. haematobium* infections through drug therapy or natural immunity and yet still have parasite eggs in their reproductive tracts. To address this issue, we directly microinjected viable *S. haematobium* eggs into the vaginal walls of female BALB/c mice. Our overall aim was to create a mouse model to study FGS.

## Methods

### Ethics statement

All animal work was conducted according to relevant U.S. and international guidelines. Specifically, all experimental procedures were carried out in accordance with the Administrative Panel on Laboratory Animal Care (APLAC) protocol and the institutional guidelines set by the Veterinary Service Center at Stanford University (Animal Welfare Assurance A3213-01 and SDA License 93-4R-00). Stanford APLAC and institutional guidelines are in compliance with the U.S. Public Health Service Policy on Humane Care and Use of Laboratory Animals. The Stanford APLAC approved the animal protocol associated with the work described in this publication.

### Mice

A total of 50 mice were used for the experiment (30 egg- and 20 vehicle-injected controls). Seven to eight week old female BALB/c mice were purchased from Jackson Laboratories and housed in the Veterinary Service Center at Stanford University.

### *S. haematobium* egg isolation

S. haematobium-infected LVG hamsters were obtained from the National Institute of Allergy and Infectious Diseases Schistosomiasis Resource Center of the National Institutes of Health. The hamsters were sacrificed at the point of maximal liver and intestinal *Schistosoma* egg levels (18 weeks post-egg injection [25], at which time livers and intestines were minced, homogenized in a Waring blender, resuspended in 1.2% NaCl containing antibiotic-antimycotic solution (100 units penicillin, 100 µg/mL streptomycin and 0.25 µg/mL amphotericin B, Sigma-Aldrich), passed through a series of stainless steel sieves with sequentially decreasing pore sizes (450 µm, 180 µm, and 100 µm), and finally retained on a 45 µm sieve. Control injections were performed using similarly prepared liver and intestine lysates from age-matched, uninfected LVG hamsters (Charles River Laboratories).

### *S. haematobium* egg injection

**Figure 1:**
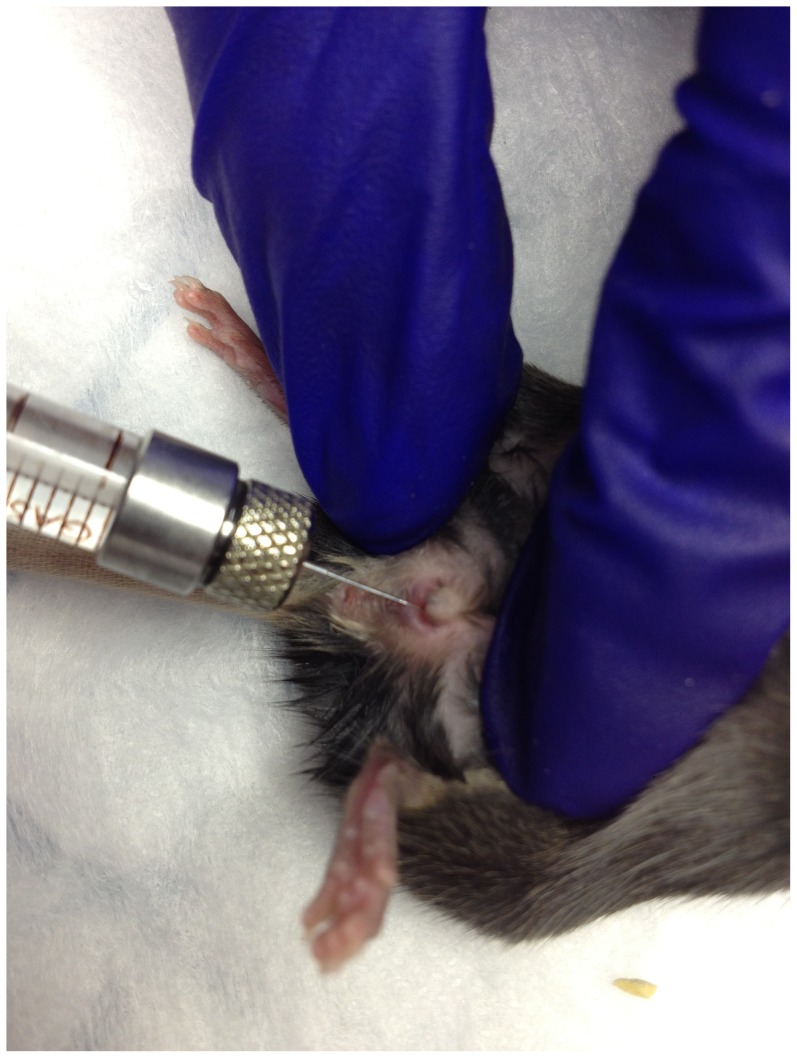
Egg injection of *S. haematobium* into mouse vaginal introitus at 6 o'clock position.

Seven to eight week old female BALB/c mice were anesthetized with isoflurane. Freshly prepared *S. haematobium* eggs (1,000 eggs in 50 µl of phosphate-buffered saline, experimental group) or uninfected hamster liver and intestinal extract (in 50 µl of phosphate-buffered saline, control group) was injected submucosally into the mouse posterior vaginal wall at 6 o'clock over 5 seconds (Figure 1). For mice undergoing sacrifice for flow cytometry and Luminex experiments, additional eggs (1,000 eggs in 50 µl of phosphate-buffered saline, experimental group) were injected at the 3, 6, and 9 o'clock positions into the mouse posterior vaginal wall. By both observation of miracidial activity and hatch testing a high proportion of viable eggs was confirmed for each batch of eggs injected. All egg injections were performed within 8–10 hours of egg isolation. (>70% of eggs from by each batch were confirmed to be viable through observation of motile miracidia within eggs and successful hatch tests).

### Voided spot on paper analysis

Voided spot on paper analysis was performed as previously described [26]. In brief, mice underwent vaginal injection with either eggs (n = 15) or control vehicle (n = 5). One week later, mice were housed singly and acclimated for one hour in cages lined with filter paper laid underneath a wire floor bottom. Animals were given *ad libitum* access to food and water-soaked sponges placed on wire cage covers. After 8 hours, each piece of filter paper was photographed under ultraviolet light to localize voided urine spots. Total spots were counted for each mouse and the average number of voids were compared between the egg- and vehicle-injected mice using two-tailed T-tests.

### Histopathologic analysis

Mice were sacrificed at serial time points 2, 4, 6 and 8 weeks after vaginal injection and the vaginas,cervices, and bladders processed for routine histology. Step sectioning was performed by alternating between discarding and analyzing 10 sequential 5 micron sections. Morphologic analyses were conducted on hematoxylin and eosin- stained sections. The entire vagina, bladder, and cervix of each mouse was processed, sectioned, and examined for pathology.

### Flow cytometry

Each mouse vagina was dissected in its entirety from the introitus up to the level of the cervix. The posterior cul de sac was separated from adjacent adipose tissue and skin with sharp dissection. The pubic bone was split and the vagina was gently removed from the pelvis by transecting it 5 mm below the cervix. Freshly excised vaginas were minced and incubated with agitation in 0.5% heat-inactivated FBS (Thermo Scientific Hyclone, IL), 20 mM HEPES pH 7, 125 U/ml (1 mg/mL) collagenase VIII (Sigma-Aldrich, Saint Louis, MO) in RPMI 1640 medium for 1 hr at 37°C [27]. The tissue was then passed through a 70 µm nylon cell strainer to remove undigested tissue and macrocellular debris. A total of 10^6^ cells/sample were treated with mouse anti-CD16/CD32 (clone 2.4G2, BioLegend, San Diego, CA) for 20 min and stained with surface markers of lymphocyte lineages [mouse anti–CD3-APC-Cy7 (clone 17A2, BD Pharmingen, San Diego, CA), anti–CD4-Pacific Blue (clone RM4-4, BioLegend), anti–CD8a-Alexa Fluor 647 (clone 53-6.7, BioLegend), anti-CCR5-PE (clone HM-CCR5, BioLegend) and anti-CXCR4-PerCP efluor 710 (clone 2B11, eBioscience, San Diego, CA)]; or surface markers of myeloid lineage [anti-F4/80-FITC (clone BM8, Biolegend), anti–CD11b-APC-Cy7 (clone M1/70, BioLegend), anti–CD11c- Pacific Blue (clone N418, BioLegend), anti-CD64 (clone X54-5/7.1, BioLegend), anti-CXCR4-PerCP efluor 710 and anti-MerTK (clone AF591, R&D Systems, Minneapolis, MN) with anti-goat-IgG-APC (R&D systems)] for 30 minutes at 4°C. Flow cytometry was performed using a BD LSRII flow cytometer and BD FACS Diva software.

### Luminex

To ascertain whether the presence of *S. haematobium* eggs would induce a systemic immune response we performed serum cytokine assays. Serum samples were assayed using a mouse 26-plex cytokine kit (Affymetrix, Santa Clara, CA) according to the manufacturer's instructions. Samples were read using a Luminex 200 (Luminex, Austin, TX) with a lower cut off of 100 beads per sample (Human Immune Monitoring Core, Stanford University). Assayed proteins analyzed included: IL-1α, IL-1 β, IL-2, IL-3, IP10, IL-4, IL-5, IL-6, IL-10, TGF-β, IL-12p40, IL-12p70, IL-17, IL-13, KC, IL-23, RANTES, IFN-γ, GM-CSF, TNF-α, G-CSF, MIP-1α, MCP-3, eotaxin, MCP-1, and VEGF.

### Statistical analysis

Flow cytometric data were analyzed using FlowJo v7.2.4 (Tree Star, Ashland, OR). An unpaired Mann-Whitney U test was used to analyze flow cytometric data and Luminex analysis between control- and egg-injected mice at each time point. Data were expressed as medians. A p value of <0.05 was considered statistically significant.

## Results

### Vaginal submucosal *S. haematobium* egg injection induces granuloma formation and maturation

**Figure 2:**
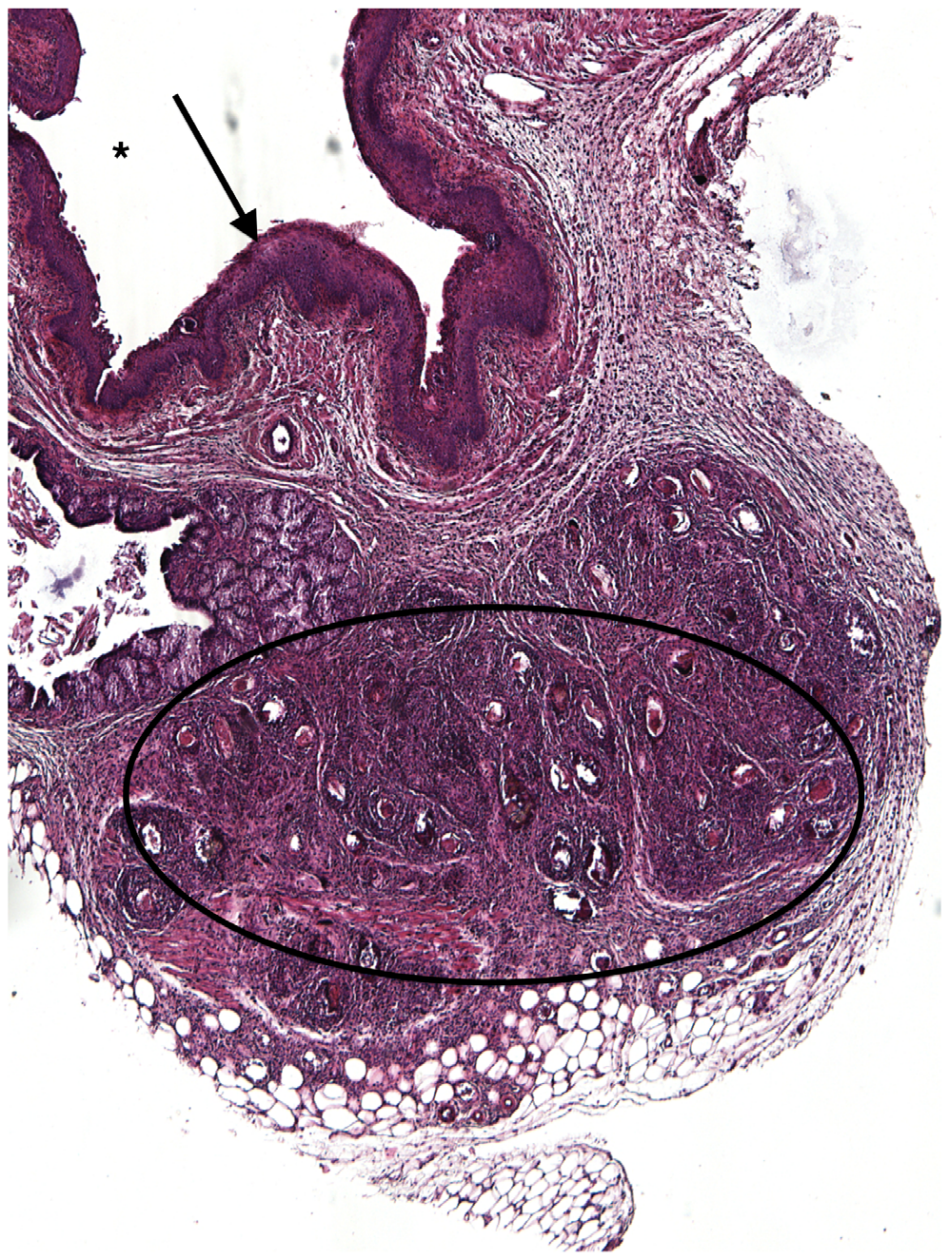
H&E Mouse vaginal walls injected with *S. haematobium* eggs post infection at 2 weeks. Injections at all time points resulted in formation of schistosome egg-based granulomata, which consist mainly of eosinophils, neutrophils, plasma cells, lymphocytes, and epithelioid cells. Asterisk = Vaginal lumen, Arrow = vaginal epithelium, Circle = granuloma.

**Figure 3:**
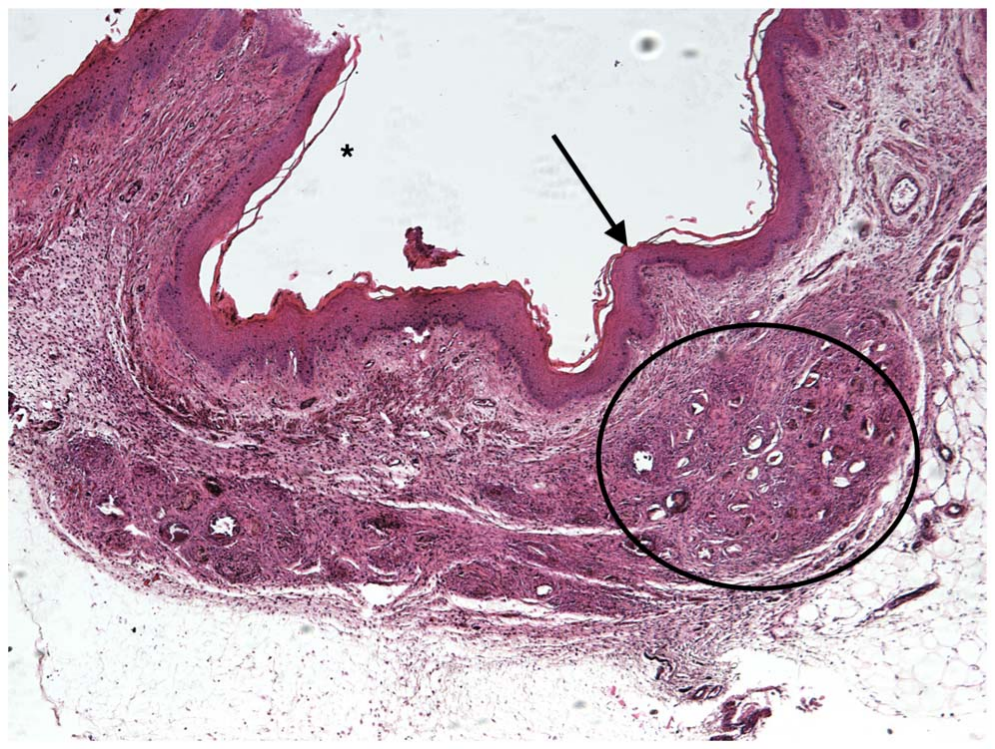
H&E Mouse vaginal walls injected with *S. haematobium* eggs post infection at 4 weeks (See description in Fig. 2.).

**Figure 4:**
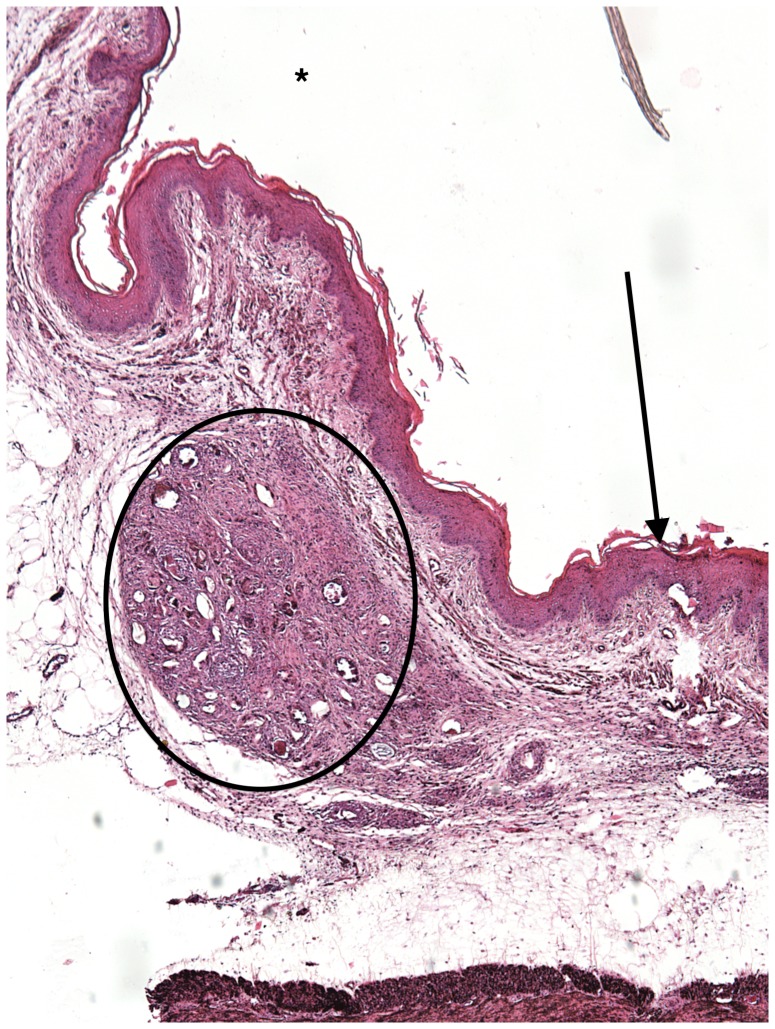
H&E Mouse vaginal walls injected with *S. haematobium* eggs post infection at 6 weeks (See description in Fig. 2.).

**Figure 5:**
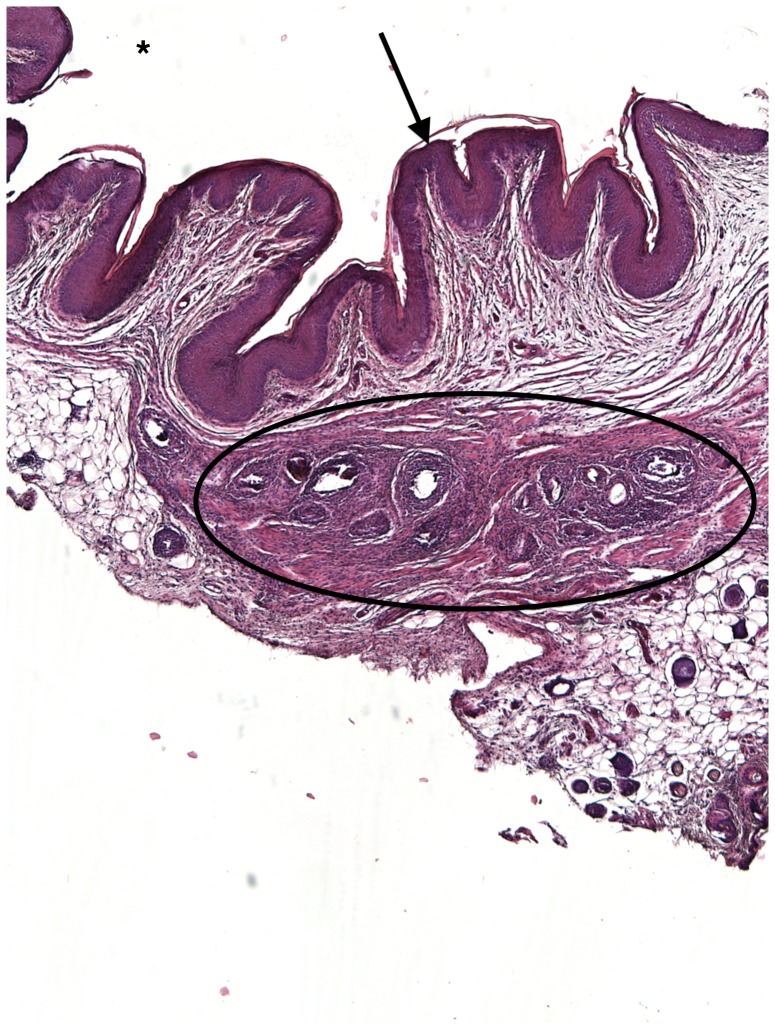
H&E Mouse vaginal walls injected with *S. haematobium* eggs post infection at 8 weeks (See description in Fig. 2.).

**Figure 6:**
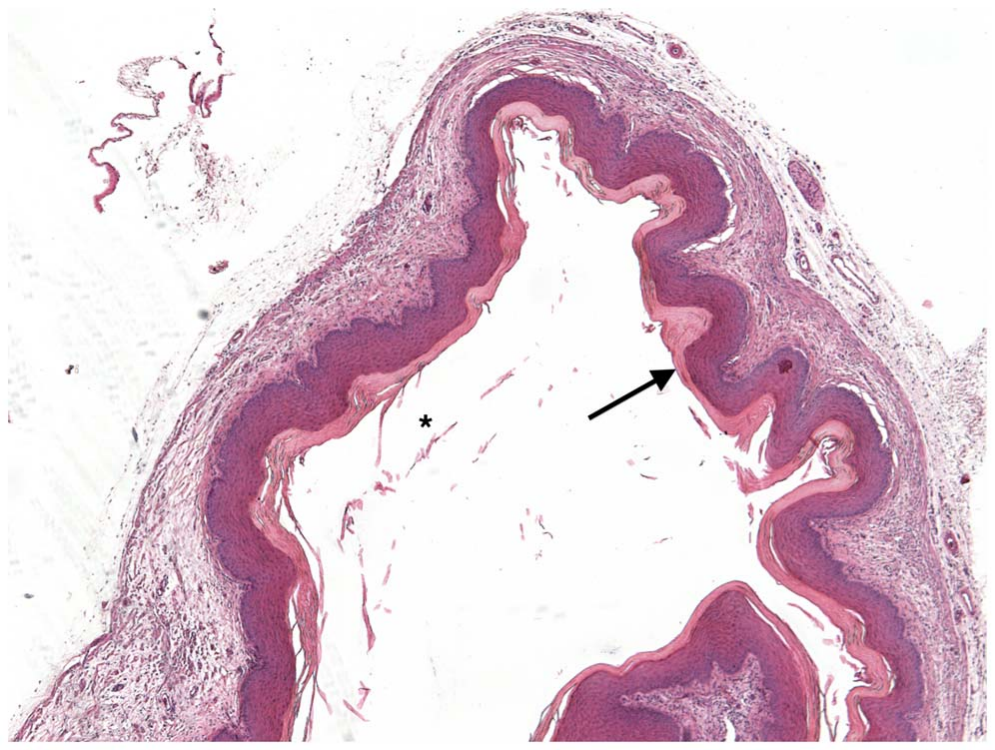
H&E Vehicle-injected mouse vaginal wall.

Over 8 weeks, the egg-associated mixed inflammatory infiltrate expanded and organized into well-defined granulomata surrounded by peripheral eosinophils and neutrophils and containing a diffuse, peripheral lymphocytic infiltrate (Figures 2–5). This is consistent with our flow cytometry data, which demonstrated an initial increase in numbers of T-cells followed by a later expansion of the macrophage pool. Intact granulomata were still present 8 weeks after egg injection. Interestingly, disruption of the vaginal mucosa, was not observed in our model. We also did not appreciate any pathology in the mouse cervix on H&E (data not shown). Accordingly, levels of keratinization and the thickness and integrity of the vaginal mucosa showed no difference in egg-injected mice compared to controls (Figure 6). Histologically we have identified intact miracidia within eggs at least two weeks after injection into mouse tissue (data not shown). This suggests that eggs remain viable for a period after injection into mouse vaginal submucosal tissues.

### Voided spot on paper

**Figure 7:**
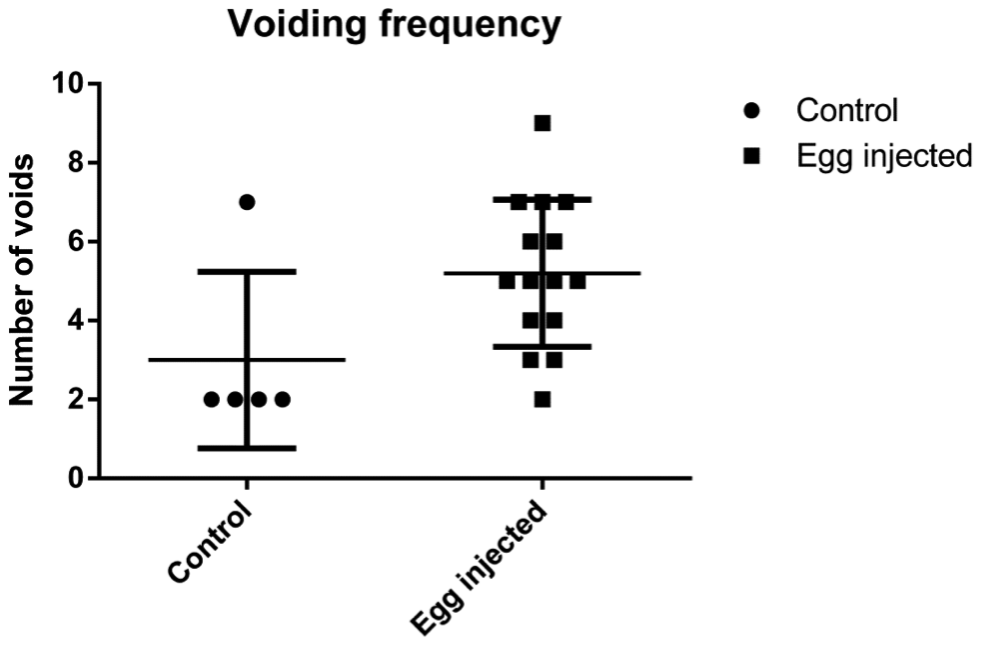
Vaginal submucosal *S. haematobium* egg injection (n = 15) increased urinary frequency compared to vehicle injection (n = 5).

Vaginal submucosal *S. haematobium* egg injection induced urinary frequency with an increase in the number of urinary voids (median number = 5) relative to vehicle-injected animals (median number = 2; p = 0.0423) (Figure 7).

### Flow cytometry

**Figure 8:**
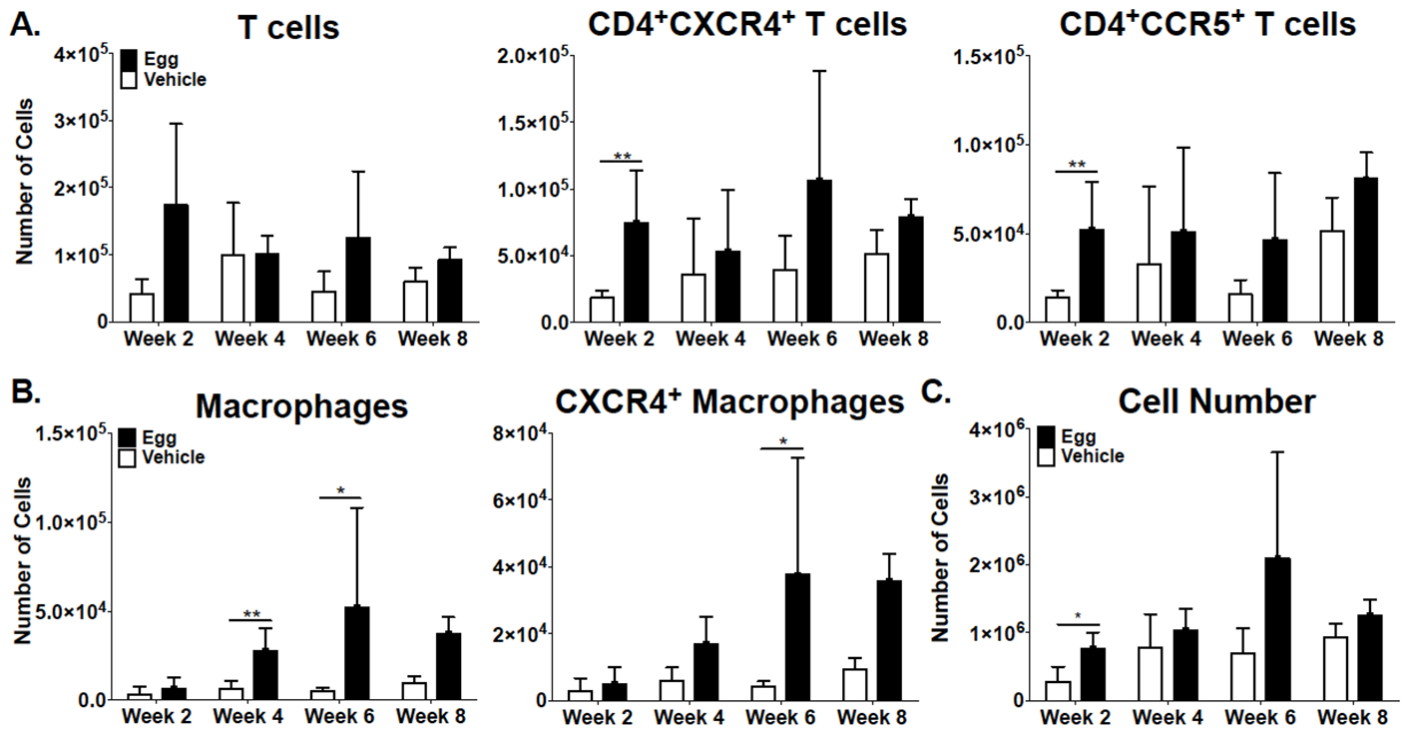
Vaginal egg injection increases numbers of HIV target cells in the vaginal submucosa. Mice injected with 3000 *S. haematobium* eggs in the posterior vaginal wall were sacrificed at 2, 4, 6, and 8 weeks post-egg injection (n = 6/group for weeks 2–6 and n = 3/group for week 8). Mice vaginas were isolated and processed for flow cytometric analysis. (A) T-cell subsets were defined by surface expression of CD3, CD4, CD8, CCR5, and CXCR4. Numbers of CD4+ CXCR4+ and CD4+ CCR5+ T cells were significantly increased in egg-injected vaginas at two weeks post-egg injection compared to vehicle-injected controls. (B) Macrophage populations were defined by the surface markers CD11b, F4/80, MerTK, CD64, and CXCR4. Numbers of total macrophages were increased significantly in egg- versus control-injected mice at 4 and 6 weeks post- injection and at 6 weeks for CXCR4+. (C) Cell numbers were counted using a hematocytometer and averaged from two separate counts per sample. Leukocytes were counted while large, morphologically distinct vaginal epithelial cells were excluded. At two weeks, compared to control-injected mice, egg-injected mice exhibited significantly increased numbers of leukocytes in vaginal tissue at 2 weeks post-egg injection.

Given the association between FGS and HIV transmission we sought to characterize potential HIV target cell populations and their HIV co-receptor (CCR5 and CXCR4) surface expression in vaginal tissue from *S. haematobium* egg-injected mice. Total T-cell subsets were defined by surface expression of CD3, and then further categorized by the surface expression of CD4, CD8, CCR5, and CXCR4. Macrophage populations were defined by the surface markers CD11b, F4/80, MerTK, and CD64, and further characterized by CXCR4 expression. Egg-injected vaginal tissue contained significantly higher numbers of both CD4+CCR5+ T cells (p = 0.0079) and CD4^+^CXCR4^+^ T cell (p = 0.0079) populations by week two post-egg injection (Figure 8A). Egg-injected vaginal tissue also had greater numbers of T cells, CD4+CXCR4+ T cells, and CD4+CCR5+ T cells throughout the 8 week time course, though these trends were not statistically significant. An increased number of macrophages expressed the HIV co-receptor CXCR4 in egg-injected mice at week 6 post-egg injection (p = 0.0173), compared to vehicle-injected mice (Figure 8B). Macrophage numbers were increased in egg-injected vaginal tissue at week 4 (p = 0.043) and 6 (p = 0.0303) compared to vehicle-injected tissue (Figure 8C).

### Luminex

RANTES protein levels were increased at 2 weeks post-egg injection (median 23.01 pg/ml) compared to vehicle-injected (median 11.23 pg/ml, p = NS). RANTES protein levels decreased at 4 weeks post egg-injection (median 13.08 pg/ml) compared to vehicle controls (median 10.5 pg/ml) p = NS. There were no differences in any other assayed cytokine in egg- versus control-injected mice at 2 and 4 weeks post-egg injectionnjection (data not shown).

## Discussion

We present a mouse model of female genital schistosomiasis amenable to the study of immune modulation and genitourinary changes that occur with *S. haematobium* egg exposure. This model did result in an increase in numbers of potential HIV target cells in egg-injected mice. The presence of *S. haematobium* eggs in the vagina did not induce significant shifts in the overall systemic immune response. We also detected an increase in urinary frequency in *S. haematobium* egg-injected mice.

Besides identifying increased urinary frequency, we also found vaginal granuloma formation in mice after *S. haematobium* egg injection as early as 2 weeks post-egg injection. The vaginal lesions we describe herein feature cellular infiltrates that differ in composition from those seen in the mouse bladder wall egg injection mouse model. In our model there is an increase in numbers of T cells at 2 weeks whereas in the bladder model there is an increase in numbers of eosinophils and B cells [24]. At 4 weeks, our model demonstrated an increase in numbers of macrophages whereas the bladder model found an increase in numbers of T cells, B cells and neutrophils [24]. We speculate that these differences exist because the resident leukocyte populations and lymphatic tissue organization of the vaginal submucosa is distinct from that of the bladder lamina propria. These differences likely guide any resulting leukocyte responses to *S. haematobium* egg exposure. Natural infection of experimental animals with *S. haematobium* cercariae can be inefficient and slow to evolve, often taking greater than 15 weeks and yielding low worm returns [28]. Bladder pathology in mice is infrequent and often is not seen until 20 weeks post-egg injection [29]. In contrast, non-human primate models of *S. haematobium* worm-based oviposition in the pelvic organs are more consistent. One study of *S. haematobium-*infected African baboons reported that their internal genitalia possessed tan, firm polypoid patches with diffuse infiltrate of eosinophils, macrophages, plasma cells and lymphocytes seen after infections of greater than 30 weeks of duration [30]. However, compared to use of experimental mice, the utilization of non-human primates in research is more expensive, fraught with more ethical concerns, and suffers from a lack of species-specific tools. To our knowledge, the work presented herein is the first mouse model to describe vaginal immune modulation by the presence of *S. haematobium* eggs. The granulomas we report are similar to those seen in human immunopathology, with recruitment of lymphocytes, macrophages, and eosinophils to egg-containing sites [31], [32]. These inflammatory cells include CD4^+^ T-cells, which are the primary cellular targets for HIV.

Given that HIV primarily infects CD4^+^ T cells and macrophages bearing the co-receptors CCR5 [33] and CXCR4 [34], we sought to characterize potential HIV target cell populations in vaginal tissue from *S. haematobium* egg-injected mice by studying these specific co-receptors. Indeed, it has been previously demonstrated that schistosomal infection elevates expression levels of CCR5 and CXCR4 on peripheral CD4+ T-cells in *Schistosoma mansoni*-infected individuals, and biopsies of FGS lesions demonstrate increased numbers of both CD4+ T cells and macrophages [18], [35]. Relative to controls, egg-injected vaginal tissue featured increased numbers of CD4^+^CCR5^+^ T cells and CD4+CXCR4+ T-cells out to 8 weeks. This could represent a shift from acute to chronic inflammation, induced in this synchronous model; however it is difficult to say with certainty that this is a chronic phenomenon.

Nevertheless, the purported causal link between FGS and increased susceptibility to HIV transmission is a hypothesis and believed to be mechanistically multifactorial. Studies of other co-infections with HIV suggest other mechanisms for an increased susceptibility to HIV transmission [36]–[39]. One study in humans co-infected with chlamydia and HIV-1 reported that HIV replication increases in association with granulocyte generation of reactive oxygen species and increases in cytokine production (based on *in vitro* assays) may impact numbers of HIV-receptive cells [36], [37]. Another study found that both native lipoprotein and synthetic lipopeptides derived from *Treponema pallidum* induced the production of HIV in a chronically infected cell line [38]. HIV-1 has also been found to utilize the host transcription factor NF-κB to drive viral gene expression in *T. pallidum* infected cells [39]. It is likely that schistosome-HIV co-infection may induce similar host inflammatory signaling cascades, and these additional mechanisms of enhanced viral replication and transmission warrant future exploration.

In addition to co-infection associations, urogenital schistosomiasis is well-known to induce genitourinary symptoms. A recent study in an *S. haematobium* endemic area of South Africa found 35% of young girls between the ages of 10–12 reported urogenital symptoms associated with urinary schistosomiasis [40]. Symptoms included increased dysuria, burning sensation in the genitals, as well as stress and urge urinary incontinence [40]. While not all symptoms were statistically significant compared to girls without urinary schistosomiasis, infected girls reported increased episodes overall [40]. In our model, *S. haematobium*-injected mice were found to show signs of urinary frequency more often than control-injected mice. Step sectioning of pelvic organs by alternating between discarding and H&E staining 10 sequential 5 micron sections demonstrated that granulomas were restricted to the vaginal submucosa and did not migrate beyond to perivesical tissues. To our knowledge, this is the first report of FGS inducing urinary frequency in the absence of *S. haematobium* eggs in the bladder. Although egg injections were administered to the posterior vaginal wall (6 o'clock) of infected mice, they were found to have an increase in the number of voids compared to controls. Several animal models have confirmed cross-organ sensitization among the lower urinary tract and gynecologic structures [41], [42]. In an induced model of endometriosis, female rats were found to have bladder inflammation and urinary bladder hypersensitivity, reflected as a decrease in micturition thresholds [41]. A different study reported that uterine inflammation in female rats causes plasma extravasation, suggesting the existence of cross-organ inflammation [42]. Viscero-visceral referral and sensitization (termed cross-organ sensitization) has recently been described to include peripheral mechanisms [43]. This is likely due to neurons from the peripheral nervous system (PNS) that converge centrally in the spinal cord with input from the viscera, skin, muscles and blood vessels. A large number of spinal neurons are receptive to visceral afferents. There are no second order spinal neurons that specifically transmit visceral signals, thus leading to convergence of both somatic and visceral inputs into the same second order neurons [44].

Besides inducing genitourinary symptoms, FGS is widely regarded as an immunomodulating infection. We assessed a large cytokine panel and did not find that FGS induced broad, systemic immunomodulation in this mouse model. RANTES was the only chemokine found to be increased in *S. haematobium* egg-injected versus control- injected vaginal tissue, however this was a non-significant trend. We believe that RANTES could possible be elevated as an acute response to *S. haematobium* eggs. RANTES has previously been described to aid in immunity against HIV-1 by competing to bind to CCR5. Sustained RANTES binding has been reported to chronically reduce cell surface levels of CCR5 [45]. A recent meta-analysis suggested that Asians with the RANTES -28G allele may have decreased susceptibility to HIV-1 infection [46]. Few studies have described the role of RANTES in schistosomiasis infection. One study reported a classification tree created from both factor analysis and risk analysis that showed high levels of TNF-α and low levels of RANTES in men were associated with a high risk of schistosomal liver fibrosis [47]. In our mouse FGS model, RANTES was found to have fallen by week 4, which coincides with the post-granuloma formation period. A decrease in RANTES could potentially cause an increase risk of HIV transmission due to weakened immunity. To our knowledge, no study to date has reported on the relationship between RANTES and FGS.

Although there are few animal models for *S. haematobium* infection in general, most existing models are of urinary schistosomiasis [30], [48]. Given the large number of available mouse-specific tools, our model may aid the further investigation of FGS. FGS results from a very complex natural history that is challenging to replicate via transdermal infection of experimental animals with cercariae, the route of infection for humans. Instead, we have injected live eggs into the vagina and have confirmed similar granulomatous pathology seen in humans.

We recognize there are limitations to this model, as it does not reproduce true disease in which ova migrate from the lumens of host blood vessels to the epithelial surface. Instead, our model generated oviposition in the vaginal submucosa, below the vaginal epithelium. Eggs were injected below the epithelial surface and did not migrate as seen in natural infection. We therefore did not find any vaginal mucosal lesions. Sandy patches on the cervix or vaginal mucosa are pathognomonic lesions associated with human FGS and are indicative of mucosal abnormalities [16]. Thus, our model is unsuitable for examining the pathobiology of sandy patches and contact bleeding associated with FGS. Another consideration is that much of human FGS pathology is seen in the human cervix [16]. Due to the technical challenge of injecting eggs into the mouse cervix we were not able to incorporate this into our model. All mice were injected at one time point and to the same depth likely because a consistent vaginal submucosal tissue plane naturally developed during the injections. We also believe this to be a limitation of our model as we were not able to study migrating eggs at various depths within the tissue. Given that our model features synchronous progression of egg-based inflammatory lesions by virtue of a single bolus injection, all lesions evolve at the same rate and as a result appear similar to each other. In this important sense the lesions that result in our model do not resemble human FGS, in which lesions are of varying chronicity depending on when oviposition has occurred. We have also injected Sepharose beads into mouse tissues and this foreign body control also does not result in epithelial alterations (data not shown).

Ultimately, a humanized animal model of FGS (including HIV co-infection) may be informative.

The exact natural history of the local immune reactions to *S. haematobium* eggs in different phases of FGS is not known, but should be explored because it will have implications for treatment schedules and in choosing the best target populations (i.e., schoolgirls versus women). The results presented herein suggest that our novel model of FGS may give insights regarding the evolution of FGS lesions. Finally, it may be amenable to the study of *S. haematobium-*induced female reproductive tract inflammation and HIV susceptibility.
