# Correction: A New Mouse Model for Female Genital Schistosomiasis

**DOI:** 10.1371/journal.pntd.0002957

**Published:** 2014-05-22

**Authors:** The PLOS Neglected Tropical Diseases Staff

The fifth author’s name is spelled incorrectly. The correct name is: Justin I. Odegaard. The correct citation is: Richardson ML, Fu C-L, Pennington LF, Honeycutt JD, Odegaard JI, et al. (2014) A New Mouse Model for Female Genital Schistosomiasis. PLoS Negl Trop Dis 8(5): e2825. doi:10.1371/journal.pntd.0002825
